# Strategies for the production of dsRNA biocontrols as alternatives to chemical pesticides

**DOI:** 10.3389/fbioe.2022.980592

**Published:** 2022-10-10

**Authors:** James Hough, John D. Howard, Stephen Brown, David E. Portwood, Peter M. Kilby, Mark J. Dickman

**Affiliations:** ^1^ Department of Chemical and Biological Engineering, University of Sheffield, Sheffield, United Kingtom; ^2^ Sheffield RNAi Screening Facility, School of Biosciences, University of Sheffield, Sheffield, United Kingtom; ^3^ Syngenta, Jealott’s Hill International Research Centre, Bracknell, United Kingdom

**Keywords:** dsRNA, RNAi, RNAi pest control, pest management, biopesticicdes

## Abstract

Current crop pest control strategies rely on insecticidal and fungicidal sprays, plant genetic resistance, transgenes and agricultural practices. However, many insects, plant viruses, and fungi have no current means of control or have developed resistance against traditional pesticides. dsRNA is emerging as a novel sustainable method of plant protection as an alternative to traditional chemical pesticides. The successful commercialisation of dsRNA based biocontrols for effective pest management strategies requires the economical production of large quantities of dsRNA combined with suitable delivery methods to ensure RNAi efficacy against the target pest. A number of methods exist for the production and delivery of dsRNA based biocontrols and here we review alternative methods currently employed and emerging new approaches for their production. Additionally, we highlight potential challenges that will need to be addressed prior to widespread adoption of dsRNA biocontrols as novel sustainable alternatives to traditional chemical pesticides.

## Introduction

### Global food security and pest management

Plant pests and pathogens are estimated to reduce crop yields by 20–40% each year, with insect pests alone already consuming anywhere from 5–20% of major grain crops (Deutsch et al., 2018), leading to reduced food security at household, national and global levels (Flood, 2010; Cerda et al., 2017; Douglas, 2018). Food demand is currently at its highest and is set to increase with global population, which is predicted to hit 9 billion within four decades (Andreev et al., 2013). The free movement of populations, combined with the effects of global warming are increasing the range of pest species and crop diseases, generating new challenges for current crop protection strategies (Hilder and Boulter, 1999; Bebber et al., 2013). Historically, a range of alternative crop protection agents have been implemented in the agricultural sector (Dubey et al., 2011). However, recently there have been several developments which threaten current global food security. Worldwide, the use of pesticides has been in decline since 2007 - largely due to stricter regulations and public opinion pressures. Whilst providing benefit by protecting crop yields and helping to ensure land use efficiency, issues with pesticide use have arisen from the appearance of resistance to existing products and the growing customer concerns about high-intensity agricultural practices (Hilder and Boulter, 1999; Zhang, 2018). Beyond the use of traditional small molecule pesticides as a “chemical” method of pest control, alternative “biological” methods of pest control have been developed, such as the spray application of toxins derived from *Bacillus thuringiensis* (*Bt*) to crops, which has been deployed for more than 60 years (Bravo et al., 2011; Heckel, 2020). However, many important insect pest species are not susceptible to these methods of control, while other previously susceptible species have developed resistance to treatments such as *Bt* toxins (Gordon and Waterhouse, 2007; Tabashnik et al., 2013; Tabashnik and Carrière, 2017).

There are several major drivers for the development of new classes of pesticides, chief among which is the economic cost of pest damage to agriculture, and particularly the increasing cost due to increasing pesticide resistance (Gould et al., 2018), with pest and pathogen damage resulting in over $100 billion worth of damage annually. Incidences of pesticide resistance have dramatically and relentlessly increased since the 1950s (Whalon et al., 2008), and the spread of the natural range of resistant insects, such as Colorado potato beetle, with climate change threatens to magnify the amount of crop damage done by these insects (Bebber, 2015). Just a one degree Celsius rise in temperatures could increase the total losses of rice, corn and wheat alone by 10–25%, with a two degree Celsius rise resulting in approximately 213 million tons of lost produce (Deutsch et al., 2018).

Current crop pest control strategies rely on insecticidal and fungicidal sprays and plant genetic resistance and/or transgenes. There is a growing demand for innovative, sustainable approaches to crop protection motivated by: an ever increasing population, climate-driven pest range expansion, community and regulatory demands, and pest resistance to traditional agro-chemicals (Savary et al., 2012).

### RNAi based crop protection

**FIGURE 1:**
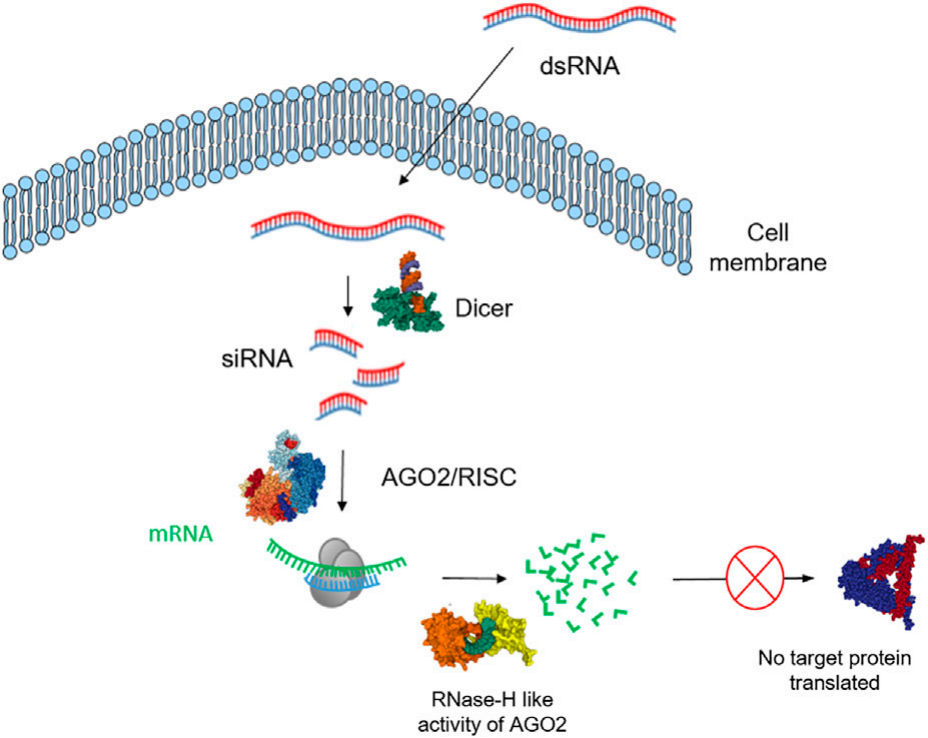
Schematic illustration of the exogeneous RNA interference (RNAi) pathway in insects. Dicer-2, cleaves long dsRNAs of either cellular or viral origin into siRNAs that mediate an endogenous or antiviral RNA interference (RNAi) response preferentially sorted to the Argonaute-2 (AGO2) RISC, which mediates sequence-specific target cleavage and degradation.

The application of double-stranded RNA (dsRNA) for the sequence specific degradation of targeted mRNA *via* RNA interference (RNAi) is emerging as an important tool for the development of novel RNA-based sustainable insect management strategies (Katoch et al., 2013; Dalaisón-Fuentes et al., 2022). The advantages and disadvantages of using different RNAi technologies to protect plants from insect pests have been reviewed (Liu et al., 2020). In addition to the regulation of endogenous gene expression, another function of the RNAi pathway in invertebrates and plants is to act as a defence mechanism, providing innate immunity against viruses that produce dsRNA (Paces et al., 2017). It is an endogenous cellular process and is a form of post-transcriptional regulation in which dsRNA directs cleavage of complementary endogenous mRNA (Price and Gatehouse, 2008), resulting in loss of protein production (*see* Figure 1). RNAi is believed to have evolved as a defence mechanism against viral RNA (Obbard et al., 2009) and to maintain the integrity of genomic DNA (Agrawal et al., 2003).

The 2006 Nobel Prize in Physiology or Medicine was awarded to Andrew Fire and Craig Mello following their pioneering work in 1998, in which this endogenous process was manipulated (Fire et al., 1998)*.* This research monitored phenotypic changes in nematode worms (*Caenorhabditis elegans)* following the introduction of exogenously produced dsRNA (Fire et al., 1998). This discovery revolutionised functional genomics studies, and further work highlighted the potential for RNAi to play a key role in the agricultural sector for the protection of crops (Baum et al., 2007; Mao et al., 2007). The RNAi mechanism can result in the degradation of target mRNA upon entry of a specific dsRNA into the cell (Zhang, 2018). Therefore, by delivering dsRNA targeting an endogenous mRNA of the intended pest, production of the encoded protein can be reduced at the post-transcriptional level. Thus, through careful selection of an essential target mRNA, this mechanism can lead to insect mortality. The sequence-specific nature of RNAi makes dsRNA an ideal candidate for further application as a species-selective insecticide and it is emerging as an important novel and sustainable insect and fungal management strategy.

Potential uses for triggering RNAi in the agricultural sector include: bio-pesticides (Baum et al., 2007; Mao et al., 2011; Zhang et al., 2017; Meng et al., 2020; Yan et al., 2020), plant virus repression (Viswanath et al., 2000; Wang et al., 2000; Mitter et al., 2017) and prevention of parasites or viral infections of pollinators (Hunter et al., 2010; Paldi et al., 2010; Garbian et al., 2012). There is also scope for using this approach to target insect vectors of human disease, for example mosquitoes or zika flies. A significant advantage of RNA-based bio-controls is the ability to target individual pest species, therefore reducing the use of non-specific pesticides (Zhang et al., 2015).

**FIGURE 2:**
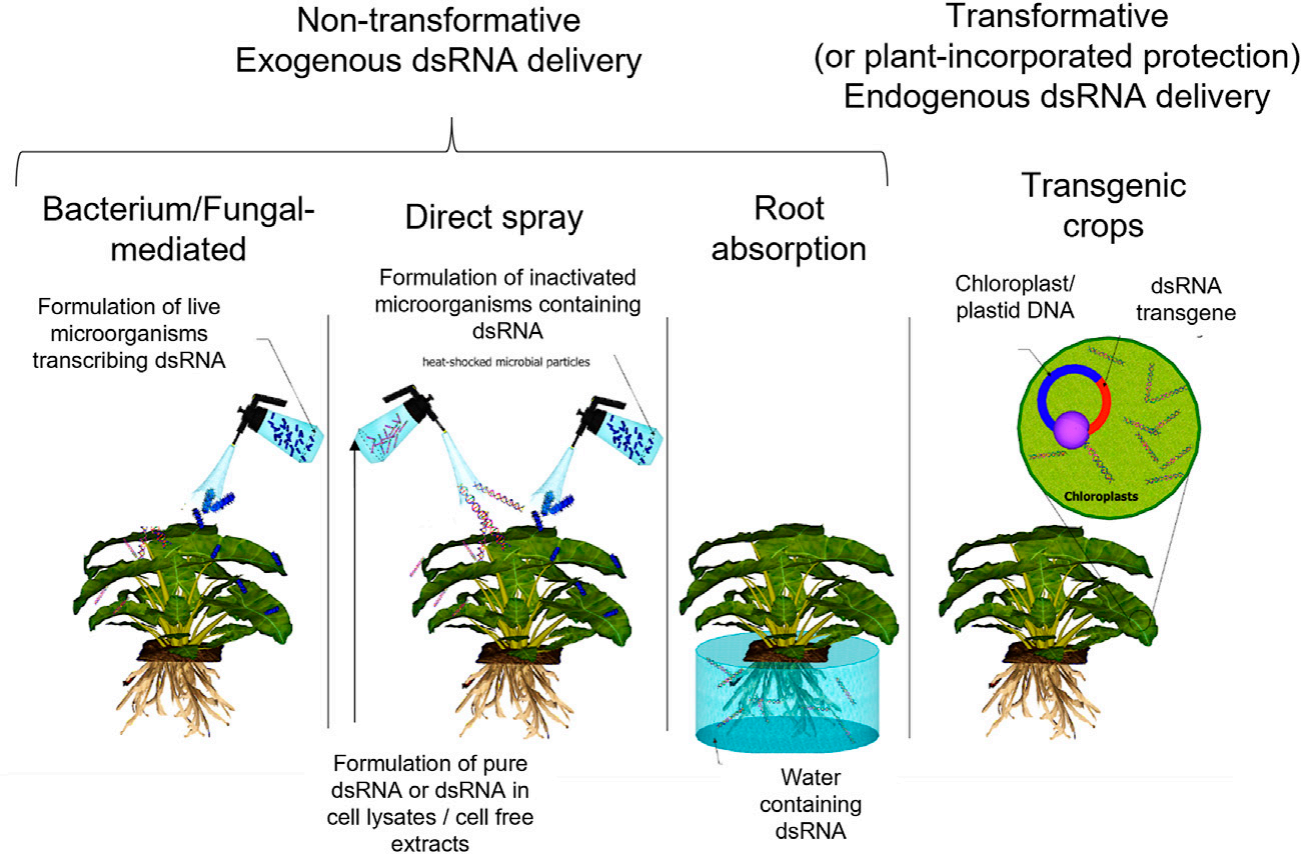
A summary of the delivery methods for endogenous and exogenous dsRNA. Non-transformative strategies which deliver exogenously synthesised dsRNA, include methods where dsRNA is synthesised *in vitro*, and those where dsRNA is synthesised and/or delivered *in vivo* in bacteria or fungi. Transformative strategies refer only to methods where the crop plant itself contains a dsRNA transgene, often in chloroplasts or other plastids.

There are several strategies for the delivery of dsRNA for pest management, all of which have suitable applications and limitations (Figure 2). Firstly, production and delivery of dsRNA can be broadly divided into transformative and non-transformative strategies. Transformative strategies involve the introduction of genes encoding insecticidal dsRNA into plants by genetic engineering, resulting in endogenous production of dsRNA within the plant (Baum et al., 2007). This is typically within plastids (chloroplasts *etc*.) which lack genes involved in plant post-transcriptional gene silencing (PTGS) such as the dsRNA-specific Dicer-like (DCL) ribonucleases. Therefore, dsRNA is accumulated in plastids within the plant without being degraded by DCL ribonucleases as it would be in the cytoplasm (Cagliari et al., 2019). However, in some commercial dsRNA insecticide products such as MON 87411 maize, the dsRNA expression cassette is chromosomally integrated rather than being within plastids (Naegeli et al., 2018). In the case of MON 87411, this is because it targets corn rootworm, which feed on the roots of the plant which lack chloroplasts. These strategies are also often referred to as plant incorporated protectants (PIPs) (Parker and Sander, 2017).

Non-transformative strategies encompass a wider range of delivery methods, though all of these involve the topical application of dsRNA synthesised (exogenously from the target crop plant) either *in vitro*, or in microorganisms (Wang et al., 2018; Yoon et al., 2018; Hashiro and Yasueda, 2022). dsRNA synthesised in microorganisms can be extracted prior to application, applied within dead microorganisms, or applied within live microorganisms. This latter strategy is referred to as bacterium-mediated or bacterially mediated RNAi, and there are several different sub-strategies encompassed within this term, which will be discussed below. It is worth clarifying that “non-transformative” specifically refers to a strategy where there is no genetic transformation of the crop plant but includes strategies where microorganisms may be genetically transformed to produce dsRNA based biocontrols.

Exogenous (i.e. non-transformative) strategies require the dsRNA to be applied to the crop, and therefore utilise a number of delivery methods. For example, similar to the current application methods used for chemical pesticides, the purified dsRNA or material containing the dsRNA can simply be sprayed directly on the crop (Tian et al., 2009; Huvenne and Smagghe, 2010; Zhu et al., 2011; Petrov and Galabov, 2012; San Miguel and Scott, 2016; Gogoi et al., 2017; Mamta and Rajam, 2017; Davis-Vogel et al., 2018; Niehl et al., 2018) (*see* Figure 2). The dsRNA can be applied by direct spraying onto the target crop plant, and these methods are collectively referred to as spray-induced gene silencing (SIGS) strategies (Koch et al., 2016), as opposed to methods involving transgene insertion into plants, known as host-induced gene silencing (HIGS) (Koch et al., 2019). Furthermore, additional successful methods of exogenous foliar application include application of formulated dsRNA (Christiaens et al., 2018), liposome encapsulated dsRNA (Castellanos et al., 2019), nanoparticle-bound siRNAs (Thairu et al., 2017), and dsRNA loaded onto clay nanosheets (Mitter et al., 2017; Jain et al., 2022). Another approach for introducing exogenous dsRNA based bio-controls is root absorption, which involves integration of the dsRNA into the irrigation systems of crops (*see* Figure 2) (Hunter et al., 2012; Ghosh et al., 2017). There are also many examples of successful delivery of insecticidal dsRNA in microorganisms, including delivery in yeast (Murphy et al., 2016), and delivery in *E. coli* (García et al., 2015; Kim et al., 2015).

With the continued development of new innovative dsRNA production methods, alternative delivery methods are now emerging. For example, the recent development of dsRNA produced in yeast allows for live bait stations to be utilised as a potential method of delivery (Duman-Scheel, 2018) and fungal synthesis of dsRNA enables live fungus to be grown on the exterior of crops, protecting them from feeding insects (Hu and Wu, 2016). In addition to these exogenous approaches, endogenous (transformative) approaches involving modifying the crop to endogenously produce insecticidal dsRNA (*see* Figure 2) (Baum et al., 2007; Mao et al., 2011) continue to be improved. The methods used and challenges associated with the delivery of dsRNA based biocontrols have been reviewed elsewhere (Terenius et al., 2011; Christiaens et al., 2020). For each of these delivery methods, there are several alternative production methods which are the focus of this review.

## Strategies for production of dsRNA for crop protection

Non-transformative methods used to produce exogenous dsRNA include *in vitro* transcription (IVT), microbial expression in bacteria or fungi, and cell-free synthesis. Conversely, transformative methods require the creation of transgenic plants (GM crops). Both methods have their advantages and limitations, with each of the production methods providing specific modes of action. Within this review the mechanism of each dsRNA production system will be described, with a focus on production yields, scalability and the current implementation status of each.

### *In vitro* transcription

**FIGURE 3:**
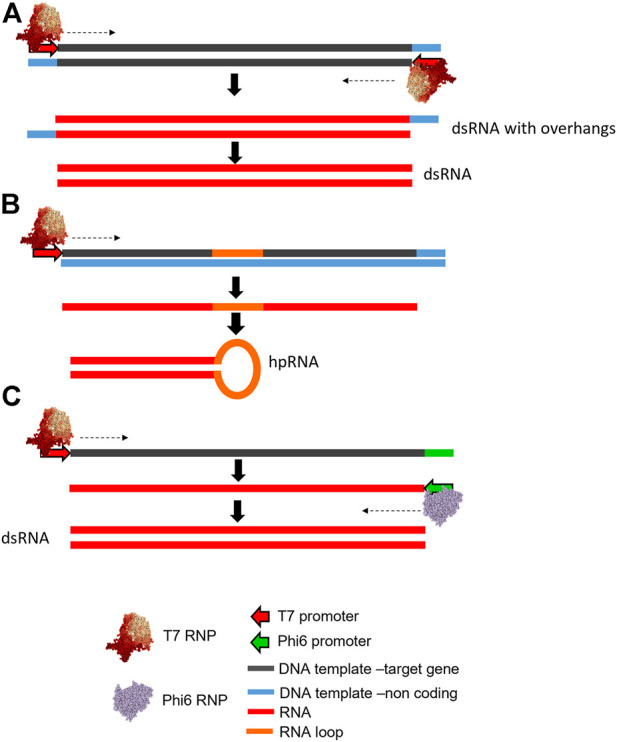
Schematic illustration of alternative DNA template designs for *in vitro* transcription of dsRNA. **(A)** A DNA template containing convergent T7 DdRp promoters flanking the target sequence is used to generate dsRNA in a single reaction with resulting 3′ overhangs that can be removed using RNase enzymes. **(B)** A DNA template using a single T7 DdRp promoter, target sequence and a sequence corresponding to an RNA loop is used to generate a long hairpin RNA (lhpRNA). **(C)** A DNA template containing a single T7 DdRp promoter site, and a ϕ6 promoter region which flank the dsRNA target sequence is used to synthesise the sense ssRNA containing the ϕ6 promoter. ϕ6 RdRP subsequently synthesises the antisense strand resulting in the production of dsRNA with blunt ends.

*In vitro* transcription (IVT) has been routinely used for the production of RNA for a wide range of molecular biology applications, including the synthesis of dsRNA for laboratory studies of RNAi in insects such as Colorado potato beetle (San Miguel and Scott, 2016), Western corn rootworm and Southern corn rootworm (Baum et al., 2007; Hu et al., 2019), Asian corn borer moth (Wang et al., 2011), spider mites (Yoon et al., 2018), brown planthoppers (Wang et al., 2018), and pea aphids (Yang et al., 2020), among many others. IVT involves the enzymatic synthesis of RNA from a DNA template in a single reaction. Most IVT reactions employ the bacteriophage T7 DNA dependant RNA polymerase (DdRp) (Wang et al., 2018; Yoon et al., 2018; Hu et al., 2019; Dumas et al., 2020; Yan et al., 2020; Yang et al., 2020). Alternative DdRp used include T3 or SP6 (Rajagopal et al., 2002; Alder et al., 2003; Skelly et al., 2003). The RNA polymerase promoter sequence required differs between T3, T7 and SP6 DdRps, as does the optimum number and arrangement of transcriptional promoters and/or terminators (*see* Figure 3). Alternative *in vitro* systems have also been utilised including a dual polymerase system using both T7 DdRp and Phi6 (ϕ6) RNA dependant RNA polymerase (RdRp) (*see* Figure 3C) (Petrov and Galabov, 2012; Levanova and Poranen, 2018; Niehl et al., 2018).

For the synthesis of dsRNA, run-off transcription is typically employed. It has previously been demonstrated that products of *in vitro* transcription by T7 DdRp may have up to three additional 5′ G residues, and 3′ overhangs of up to 12 additional nucleotides at the 3′ end (n + 12) (Gholamalipour et al., 2018). The same study also proposed a mechanism by which T7 polymerase switches from the DNA to bind to the end of the RNA product and continues synthesis of further RNA using the initial RNA product as a template, in a process termed primer extension. While deliberate utilisation of this mechanism in the dual polymerase T7-ϕ6 system produces effective insecticidal dsRNA, random primer extension results in double-stranded hairpin regions at the end of single stranded transcripts, which will subsequently affect the binding of complimentary ssRNA transcripts into dsRNA, and how key RNAi pathway enzymes such as Dicer effectively process these dsRNA substrates. IVT reactions can also produce by-products comprised of short products formed by inefficient transcription, often referred to as “shortmers.” Additionally long contaminants or “longmers” may arise, either from promoter-less transcription, extension by priming using RNA-dependant RNA synthesis (Gholamalipour et al., 2018, 2019), or as a result of the formation of multimers or aggregates of dsRNA (Nwokeoji et al., 2019). However, short single-stranded overhangs are easily removed by incubation with a ssRNA-specific ribonucleases such as RNase T1, in order to produce blunt end fragments. This ensures the purity of the product is high and minimal purification steps are required to remove excess NTPs, template DNA and protein contaminants (Losick, 1972; Thermo Fisher Scientific, 2009). This strategy is well characterised, efficient, and robust, making it an ideal method for producing insecticidal dsRNA.

Synthesis of dsRNA by IVT requires a DNA template of the target gene region conforming to one of the designs illustrated in Figure 3. The most widely exploited design (*see* Figure 3A) consists of the target sequence for the target gene, flanked by two convergent (i.e. facing each other in opposite directions) 5′ RNA polymerase promoter sites (Wang et al., 2018; Yoon et al., 2018; Dumas et al., 2020; Yan et al., 2020). This DNA template transcribes both sense and antisense strands which anneal rapidly within the same reaction vessel to produce dsRNA (Hu et al., 2016). However, sense and anti-sense strand synthesis is not always equal and therefore, dsRNA yield is governed by the least transcribed strand. Inefficient transcription of one strand will produce an excess of one of the component ssRNAs, and subsequently cause inefficient production of dsRNA. Additionally, promoter-less transcription, in which transcribed RNA folds back on itself to prime its own RNA-templated extension (Gholamalipour et al., 2019), creates aberrant transcripts, also reducing the efficiency of dsRNA production.

To circumvent this issue, two separate DNA templates may be generated, each of which contain a single 5′ RNA promoter region upstream of either the sense or anti-sense sequence. Sense and anti-sense ssRNAs are synthesised separately, and subsequently combined in equimolar amounts to generate dsRNA. The separate synthesis eliminates the issue of unequal synthesis of the corresponding ssRNAs. This method has been used to ensure production of high-quality dsRNA for downstream RNAi applications in insects and flatworms, and to produce separate sense and antisense strands individually with both SP6 and T7 promoter systems (Rajagopal et al., 2002; Skelly et al., 2003).

An alternative approach for synthesising dsRNA biocontrols using IVT requires only a single RNA polymerase promoter region at the 5′ end of the DNA template. The DNA template has both sense and antisense target sequences on the same strand separated by a short spacer sequence which forms a hairpin loop post-transcription, thus generating long hairpin RNA (lhpRNA) (*see* Figure 3B). This DNA template is rarely employed for *in vitro* synthesis of long dsRNA and is utilised only when hairpin templates are cloned from pre-synthesised plasmid DNA (Alder et al., 2003; Urquhart et al., 2015).

In addition to the IVT methods described, alternative hybrid approaches using two different RNA polymerases have been used. For example, a system has been developed in which the DNA template contains a single T7 RNA polymerase promoter site, and a ϕ6 RNA polymerase promoter (AAAAAAAAGG), which flank the dsRNA target sequence. Following the synthesis of the sense ssRNA by T7 DdRp, the ϕ6 RdRp is subsequently able to recognise the RNA polymerase promoter sequence and synthesises an entire complimentary antisense ssRNA strand *in situ*, to form the dsRNA (Niehl et al., 2018) (*see* Figure 3C). The advantage of using the hybrid system over other methods is the high purity of the dsRNA product, which has true blunt ends and lacks the potential overhangs which are produced by other polymerases such as T7 (Makeyev and Bamford, 2000) or other template configurations, as previously described. Notably, convergent promoters (Figure 3A) are the most widely employed templates for IVT synthesis, and have been widely adopted for screening for potential RNAi targets (Wang et al., 2018; Yoon et al., 2018; Hu et al., 2019). This is largely due to the simplicity of construct design and synthesis, which can be achieved by generation of a DNA template using PCR reactions with sequence specific primers typically conjugated to a 5′ T7 RNA polymerase promoter sequence (5′-TAATACGACTCACTATAG-[Target primer]-3′), which following purification can be used to produce dsRNA of the target (Wang et al., 2018; Hu et al., 2019; Dumas et al., 2020; Yan et al., 2020; Yang et al., 2020). This relatively short method produces high purity dsRNA, enabling the rapid analysis of large numbers of potential new targets. Current commercial high yield IVT reactions are widely available and typically generate approximately 50–100 µg of RNA per reaction. Large-scale IVT is limited but commercially available (http://genolution.co.kr/agrorna/service-overview/).

There are several advantages of *in vitro* transcription over other scalable systems, including the relatively simple purification of the dsRNA product when compared to *in vivo* production systems. Agricultural products do not necessarily require the high standard of purity required for oligonucleotide/siRNA therapeutics, although some delivery methods may be inhibited by impurities. Many studies have shown the short life span of naked dsRNA in the environment, with improvements in RNAi activity achieved when the dsRNA is encapsulated and therefore protected from environmental nucleases (Aalto et al., 2007; Lundgren and Duan, 2013; Roberts et al., 2015; Ghosh et al., 2017; Leeuwen et al., 2019; Bachman et al., 2020). In laboratory studies, dsRNA produced enzymatically has been proven effective against numerous target species through either injection and/or ingestion, including relevant crop pest insects such as Colorado potato beetle (San Miguel and Scott, 2016; Dumas et al., 2020), Western corn rootworm (Baum et al., 2007; Hu et al., 2019), soybean aphid (Yan et al., 2020), spider mite (Yoon et al., 2018), brown plant hopper (Wang et al., 2018), pea aphid (Yang et al., 2020) and grain aphid (Liu et al., 2021). In addition, larger scale field trials of dsRNA produced enzymatically have also demonstrated RNAi efficacy (Vogel et al., 2019).

### Cell-free expression systems

Cell-free systems for *in vitro* protein expression (also referred to as *in vitro* translation, or cell-free protein expression) have been widely employed to rapidly express and manufacture small amounts of functional proteins (Kigawa et al., 1999; Shimizu et al., 2001; Zimmerman et al., 2014). Cell-free coupled transcription–translation systems utilise cell lysates to transcribe mRNA coupled to protein translation *in vitro*. The cell-free extracts are optimised to contain most of the cellular cytoplasmic components required for transcription and translation. The first known cell-free extracts capable of supporting translation were made from *E. coli* prior to the development of eukaryotic *in vitro* translational systems including lysates prepared from insect embryos (Haley et al., 2003).

**FIGURE 4:**
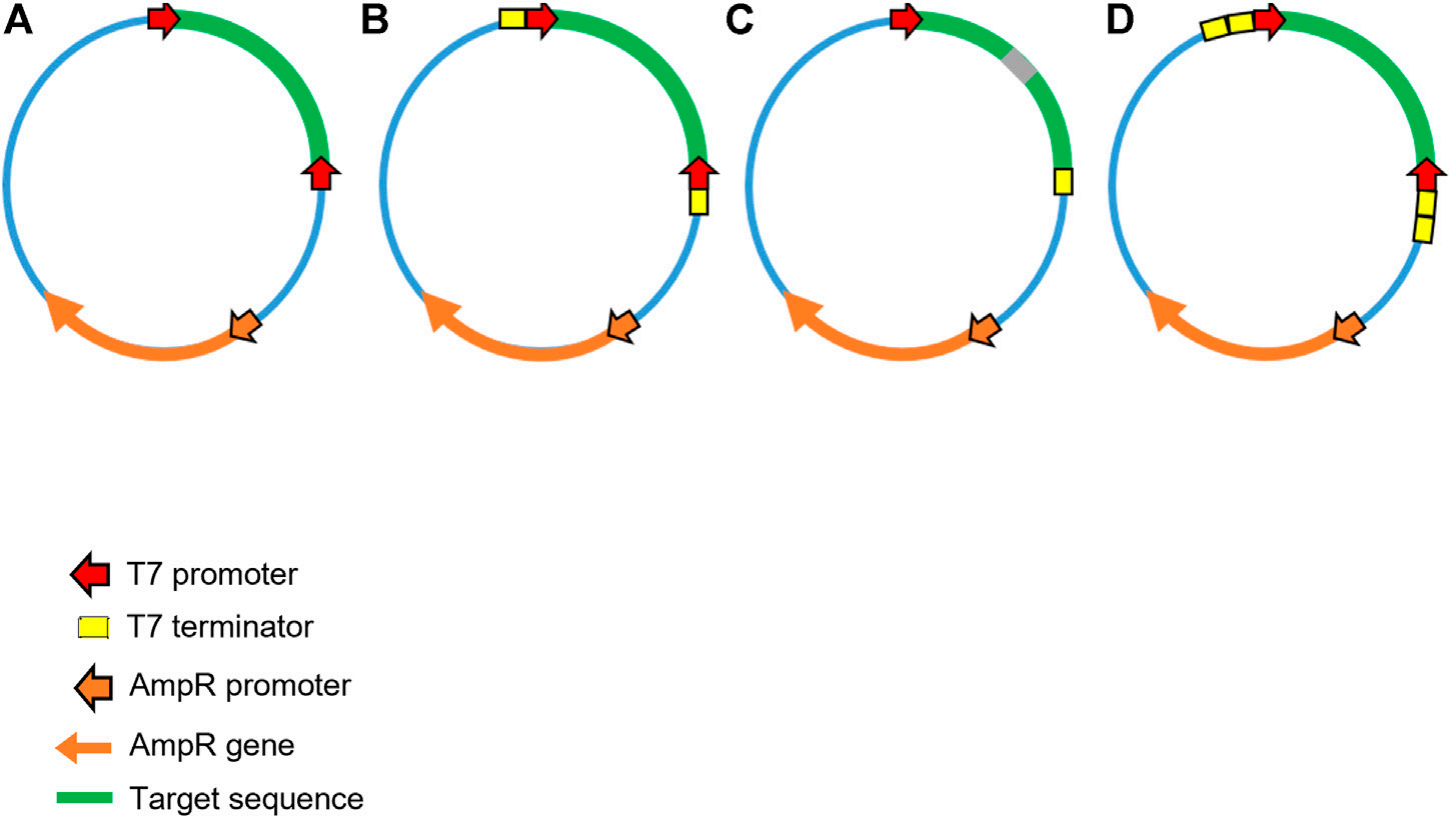
Schematic illustration of alternative plasmid DNA template designs for production of dsRNA in microbial cells. A range of alternative plasmids have been utilised for production in microbial cells. **(A)** Convergent T7 DdRp promoters flanking the target sequence without transcriptional terminators. **(B)** Convergent T7 DdRp promoters flanking the target with transcriptional terminator sequences outside of the T7 promoters. **(C)** Single T7 DdRp promoter prior to the target, a sequence corresponding to an RNA loop, the antisense target sequence and finally a T7 terminator sequence used to generate a long hairpin RNA (lhpRNA). **(D)** Convergent T7 DdRp promoters flanking the target sequence with two transcriptional terminator sequences outside of the T7 RNA polymerase promoters.

Cell free synthesis has several advantages over *in vivo* production systems. For example, the elimination of the ancillary processes required for cell viability and growth, enables all of the RNA polymerase activity and the entire pool of ribonucleotides in the reaction mixture to be utilised entirely for transcription of the dsRNA product. The absence of a cell wall generates a system that can be actively monitored with reduced sample preparation (Kanter et al., 2007; Carlson et al., 2012; Garamella et al., 2016; Fujiwara et al., 2017). Cell-free systems for the large-scale production of dsRNA focus on *in vitro* transcription from DNA templates and have been developed and commercialised by GreenLight Biosciences (https://www.greenlightbiosciences.com/in-the-pipeline-colorado-potato-beetle/). Their GreenWorx system is a cell-free platform technology that offers large scale production of dsRNA at low cost. The dsRNA is generated *via* enzymatic synthesis, with all the components required for RNA synthesis present in the cell-free system. Cell-free systems can generate hpRNA and dsRNA with a variety of potential different polymerases, using linear DNA templates similar to those used in IVT systems (*see* Figure 3) or alternatively plasmid DNA templates similar to those used in *in vivo* systems (Figure 4). Cell free systems are scalable and able to generate high yields, with production costs proposed to be as little as $0.5/G (Maxwell et al., 2018). The system also allows for the rapid change of dsRNA targets, as the DNA template is added in the final stage of the system. Potentially, it is possible to produce multiple dsRNA targets within the same reaction vessel. This would allow for the one-step synthesis of a range of dsRNA biocontrols which consist of dsRNAs targeting multiple targets within the same species for increased efficacy, or target multiple species specifically, to deal with multi-pest infestations.

### Production of dsRNA in microbial systems

#### Production of dsRNA in *E. coli*

*Escherichia coli (E. coli)* has been the widely adopted “cell factory” for production of recombinant proteins for many years. This well understood organism has high transformation efficiencies and methods of culturing it are relatively simple, inexpensive and scalable, therefore making it an ideal host for the production of biomolecules (Terpe, 2006). In addition, *E. coli* is amenable to genetic manipulation and has been widely engineered to enhance protein and biochemical production (Sørensen and Mortensen, 2005; Chen et al., 2013). Many of the methods used for recombinant protein production in *E. coli* have been adopted for the production of dsRNA (Timmons et al., 2001; Nwokeoji et al., 2017). T7 DdRp is one of the most widely utilised expression systems for the production of proteins. It is extremely well characterised, with numerous genetically engineered *E. coli* strains commercially available to suit the desired recombinant product. This availability reduces optimisation time and cost for small scale laboratory work, and provides a cost effective starting point for many research laboratories to generate larger quantities of dsRNA as an alternative to IVT (Chamberlin et al., 1970; Tunitskaya and Kochetkov, 2002; Terpe, 2006; Tegel et al., 2011).

The production of dsRNA in *E. coli* has predominantly used the bacterial strain HT115 (DE3) (Hull and Timmons, 2004; Ongvarrasopone et al., 2007; Mao et al., 2011; Posiri et al., 2013; García et al., 2015; Nwokeoji et al., 2016; Ahn et al., 2019; Bento et al., 2020; Meng et al., 2020). The HT115 cell line is of K12 lineage and was generated following the chromosomal deletion of the RNase III gene (*rnc*) to generate an RNase III deficient strain (Takiff et al., 1989). Further genetic manipulation was performed by lysogenization of the HT115 strain to insert the T7 DdRP within its genome, under the control of an IPTG inducible promoter (Timmons et al., 2001). Several alternative *E. coli* strains which are RNase III deficient have been developed. In 2009 Yin *et al.*, produced an RNase III deficient strain (M-JM109-YLac) of the common JM109-LacY (DE3) bacteria using Red proteins to knock out the *rnc* gene (Yin et al., 2009). Production of dsRNA was shown to be increased when compared to HT115 cells containing the same plasmid. It was proposed that the increase in yield was due to the strain harbouring *end*A1 and *rec*A1 genes which increase exogenous plasmid stability and produce more stable dsRNA respectively (Yin et al., 2009). Recently Ma *et al.*, produced a BL21(DE3) RNase III deficient strain by using a no-SCAR CRISPR system to efficiently knock out *rnc*. This strain demonstrated an improvement in dsRNA production yield when compared to HT115 cells (Ma et al., 2020).

The workflows adopted for production of dsRNA in *E. coli* are typical of those used for the production of recombinant proteins. Following the transformation of *E. coli* with a plasmid harbouring a dsRNA sequence which targets a mRNA of interest under the control of T7 RNA polymerase promoters, cells are then grown to exponential phase and induced for around 4–6 h. Cells are then harvested, lysed and dsRNA subsequently purified. Like IVT, there are a variety of plasmid construct designs which can be utilised to generate varying forms of dsRNA (hpRNA and linear dsRNA), all of which currently utilise T7 DdRp (*see* Figure 4). Yields from small scale shake-flask production vary but typical reported yields are <20 µg/10^8^ cells (Nwokeoji et al., 2016).

Initial plasmid DNA constructs consisted of two opposing convergent T7 promoters (face to face), flanking the dsRNA sequence (*see* Figure 4A) as described for *in vitro* transcription. The L4440 plasmid, which lacks transcriptional terminators, first designed and used by Timmons and Fire in 1998, has been widely employed for microbial expression of dsRNA and was utilised in early RNAi papers for delivery of dsRNA to *C. elegans* in *E. coli* (Timmons et al., 2001)*.* Alterations to the initial plasmid design have been adopted. The use of T7 terminators just outside of the T7 promoters suggested that this diminished the effectiveness of initiating RNAi *via* feeding in *C. elegans* (Kamath et al., 2000). In contrast, Sturm et al. indicated that the efficiency of RNAi was improved by the incorporation of terminators (Sturm et al., 2018). Alternative plasmid designs have also utilised dual terminators which improve termination efficiency and therefore increase dsRNA yield, and this strategy enabled effective RNAi effects in *A. thaliana* (Baum et al., 2014). The plasmid constructs described by Chen et al., (Chen et al., 2019) produced hpRNA, using a single T7 promoter, in front of the sense-loop-antisense sequence, subsequently followed by two sequential terminators similar to the arrangement in Figure 4C. These hpRNA-coding plasmid constructs produced twice as much dsRNA compared to a construct expressing dsRNA targeting the same sequence (*see* Figure 4D). Yields from *E. coli* HT115 cells transformed with the L4440 plasmid vary dependant on culture method, dsRNA sequence, and length. Fed batch cultures have produced up to 182 mg/L of dsRNA (Papić et al., 2018), making the production cost per mg of dsRNA less expensive when routinely performed, compared to IVT. Other combinations of vector and dsRNA sequence vary in yield when also expressed in the HT115 strain, for example a pET3a-Pro construct yielded 30 mg/L dsRNA (Ongvarrasopone et al., 2007) and a pLitmus28i construct produced 20 mg/L (Ramesh Kumar et al., 2016).

Insecticidal dsRNA expressed in *E. coli* has been utilised to induce RNAi or mortality as a result of RNAi in various species. This includes purification from *E. coli* and injection to induce RNAi in Asian lady beetle (Ma et al., 2020), and aquatic feeding of *Aedes* mosquitoes with *E. coli* lysate containing insecticidal dsRNA to induce mortality (Lopez et al., 2019). dsRNA expressed in *E. coli* and then purified prior to application, has also been used to induce RNAi in *Arabidopsis* plants (Chen et al., 2019).

#### Production of dsRNA in *Pseudomonas syringae*

In addition to *E. coli*, alternative production systems have been developed. These include a novel bacteriophage-based dsRNA production system, utilising ϕ6 RdRp to synthesise dsRNA from ssRNA templates in *Pseudomonas syringae* (Aalto et al., 2007)*.* The system involves transfecting *P. syringae* with three plasmids: one containing a constitutively expressed T7 DdRp; a second expressing the L genes of the bacteriophage ϕ6, which encode for the RdRp and capsid packaging genes; and a final plasmid containing a single stranded RNA sequence downstream of a T7 DdRp promoter, followed by a 3ʹ antisense ϕ6 RdRp promoter region.

This system has similarities to the hybrid T7-ϕ6 IVT system, however, no dsRNA is produced in the cytoplasm of the cell. ssRNA produced by T7 DdRp is transported into capsids produced by the L genes of the ϕ6 bacteriophage on the second plasmid. Housed within the capsid is the ϕ6 RdRp, which recognises the ϕ6 promoter region on the ssRNA, propagating synthesis of the complimentary antisense RNA strand, subsequently forming a dsRNA duplex. This method encapsulates dsRNA within a protective layer against endogenous RNases. dsRNA production in both small batches and in larger scale fermenters was performed, and it was established that the combination of these three plasmids within the host cell produces highly specific dsRNA in quantities of ∼1.6 mg of dsRNA/g of wet cells (Aalto et al., 2007). However, the system was unstable and inefficient in production when numerous RNA sequences were analysed (Niehl et al., 2018). A more stable and efficient system was later developed with the introduction of other ϕ6 genes which helped to stabilise plasmids within the host cells (Niehl et al., 2018). This system increased reproducibility and titre of dsRNA across a wider range of target sequences. Successful viral suppression was also reported when targeting the Tobacco Mosaic Virus (TMV), resulting in propagation suppression when exogenous dsRNA produced by *P. syringae* was applied topically (Niehl et al., 2018).

The scalability of production, and the ability to protectively encapsulate the dsRNA are key advantages to dsRNA bio-synthesis in *P. syringae*. However, the dsRNA yields achieved with this system (7 mg/L of cells at a density of 4 × 10^9^ cells/ml) (Niehl et al., 2018) are lower compared to other microbial systems e.g. 182 mg/L was obtained from *E. coli* fed batch cultures (Papić et al., 2018) In addition, *P. syringae* cultures require much longer production times, largely due to the requirement for three transfections, and the slower growth rate of this species, which has a doubling time of 1.53 h (Young et al., 1977).

#### Production of dsRNA in *Corynebacterium glutamicum*

*C. glutamicum* has recently been studied as a novel model organism for production of insecticidal dsRNA. *C. glutamicum* is a well characterised microbe capable of recombinantly producing high yields of amino acids such as L-glutamate (Ikeda and Takeno, 2013) and has recently been reported to be capable of producing large quantities of RNA (Hashiro et al., 2019c; 2019b; 2019a). These studies highlight many advantages of the bacterium as a cell factory, including its low-cost culture methods, non-pathogenicity, and it being safe and robust in large scale fermentation. This combination of traits and historical use, make it an ideal candidate for industrial RNAi applications.

An RNase III deficient *C. glutamicum* strain (2256LΔ*rnc)* was generated *via* knockout of the *rnc* gene from strain 2256L (Hashiro et al., 2019a) – which was shown not to be critical to the survival of the organism (Maeda et al., 2016). Recombinant plasmids for production of dsRNA were designed which replicated those seen in *E. coli* systems. Construct design was synergistic to the L4440 plasmid, with convergent synthetic strong F1 promoters from the corynephage BFK20 replacing the T7 RNA polymerase promoters (Figure 3A). These flanked a 380 bp homologue of the *diap1* gene of *H. vigintioctopunctata*. In a 30-h culture 75 mg/L of target dsRNA was isolated. Heat-sterilised *C. glutamicum* containing recombinantly produced insecticidal dsRNA were used in *H. vigintioctopunctata* feeding assays, and ingestion of the dsRNA-containing bacteria by the insects resulted in a significant decrease in leaf consumption and suppression in insect weight gain (Hashiro et al., 2019a).

Recently, higher yields of over 1 g/L of dsRNA targeting the *H. vigintioctopunctata* gene *diap1* were achieved in *C. glutamicum* using a similar convergent promoter arrangement, though utilising T7 RNA polymerase and the associated promoters, supplied by co-transfection of the pVC7T7pol1 plasmid (Hashiro et al., 2021). Ethanol-sterilised *C. glutamicum* containing recombinantly produced *diap1*-targeting dsRNA were again used in *H. vigintioctopunctata* feeding assays, and once more ingestion of the dsRNA-containing bacteria significantly reduced larval weight gain in a 48-h period. These studies demonstrate great promise for the industrial scale production of RNAi insecticide products in *C. glutamicum*.

#### Production of dsRNA in yeast

The potential for expression of dsRNA in yeast was demonstrated with the feeding of live genetically modified *S. cerevisiae* INVSc1 strain to fruit flies (*D. suzukii*) (Murphy et al., 2016). This study showed the potential of a new and novel application method to be implemented into the agricultural sector–live bait feeding. Previously, bait-fed live yeast has been presented for pest management in berry crops. However, this is the first study to provide a proof of concept for triggering RNAi *via* fed yeast (Murphy et al., 2016). Plasmids producing hpRNA products targeting mRNAs whose degradation is known to cause lower motility in the target species were constructed. Plasmids were of a relatively simple design and constitutively produced dsRNA, with no induction being necessary. RNAi effect was observed in *D. suzukii*, but not in other target species. Production of dsRNA in other live fungi–besides yeast–which parasitise insects has also been examined as a combined production/delivery method (*see* below).

A more recent study by Alvarez-Sanchez, et al., produced dsRNA in the yeast strain *Y. lipolytica,* targeting white spot syndrome virus of the white legged shrimp *Litopenaeus vannamei* (Álvarez-Sánchez et al., 2018)*.* dsRNA was isolated and injected into the host species. Yields of dsRNA were <182 ng/L which is low when compared to other *in vivo* systems, however, yeast has several advantages. Common baker’s yeast (*S. cerevisiae*) is supplied throughout the world as a dried product therefore, applications in live feeding stations are particularly useful in developing countries with warm climates, where cold transport is required to ensure long-term stability of dsRNA in transit (Murphy et al., 2016) but may not be available. Dried packet yeast is easily transported at a wide range of ambient temperatures, removing the limitation of cold storage availability for application of dsRNA biocontrols in warmer climates.

The fact that yeast lack the RNAi machinery used to process dsRNA is also advantageous (Zhong et al., 2019). However, in contrast to other systems they require little genetic manipulation such as the removal of ribonuclease genes (which can hinder growth rates) (Hofmann and Miller, 1977), therefore, ensuring accumulation of synthesised dsRNA. These factors, and the fact that yeast is biologically safe for human consumption, demonstrate that yeast offers an alternative system for the production and application of dsRNA based biocontrols.

#### Production of dsRNA in *Bacillus thuringiensis*

In recent years, there have been a small number of studies which have exploited *Bacillus thuringiensis* as a host bacterial production system for the expression of dsRNA (Park et al., 2020, 2021; Jiang et al., 2021). *Bacillus thuringiensis* are soil dwelling bacteria, and are also entomopathogens i.e. pathogenic to insects. In both studies by Park et al. dsRNA was expressed to target sacbrood virus (SBV), a ssRNA virus which threatens Asian Honey Bee populations. Jiang et al. produced dsRNA targeting arginine kinase in *Plutella xylostella* (diamondback both), a major pest of *Brassica* crops (Park et al., 2020, 2021). In all cases, dsRNA was expressed using convergent promoters surrounding a target dsRNA. Park et al., used *cyt*1Aa promoters, whereas Jiang et al. used Pro3α promoters.

The studies by Park et al. involved the extraction of dsRNA from the bacteria after expression in culture. Neither study provides details on the yield of dsRNA so how the efficiency of dsRNA production in *Bacillus thuringiensis* compares to other microbial systems is unknown. In contrast, Jiang et al. directly applied the live expression culture to the surface of leaves in the lab. It was determined that the intrinsic rate of increase and net reproductive rate of the insects treated with a 9:1 ratio of wildtype bacteria to bacteria expressing dsRNA were lower than those of the population treated with either wildtype bacteria alone or dsRNA-expressing bacteria alone. A similar approach was previously utilised in which *Bacillus thuringiensis* were delivered alongside an *E. coli*-expressed dsRNA targeting the immune related *Sl102* gene in *Spodoptera littoralis* (Egyptian cotton leafworm), resulting in enhanced mortality compared to the application of *Bacillus thuringiensis* alone (Caccia et al., 2020).

The minimal purification and formulation costs associated with this latter method make it an attractive approach for the joint production and delivery of a dsRNA biocontrol. However, no live transgenic *Bacillus thuringiensis* for insect pest management has been approved by regulatory bodies. The use of *Bacillus thuringiensis* as a dsRNA biocontrol expression system is in its infancy compared to other more well established systems, and further research into its viability as a practical insect control strategy is required.

#### Protection or encapsulation of dsRNA

The direct application of dsRNA in the agricultural sector has a number of limitations associated with product degradation by UV and environmental RNases which may limit overall efficacy when applied directly without formulation to protect the dsRNA (San Miguel and Scott, 2016; Leeuwen et al., 2019; Bachman et al., 2020). A potential method for protecting ssRNA and dsRNA products is the coexpression of proteins that will bind to and protect the RNA. Similar approaches have been utilised to produce and purify short hairpin dsRNA in *E. coli* (Huang et al., 2013). Co-expression of a recombinant His-tagged p19 protein and a long hairpin RNA containing sense and antisense sequences of the target mRNA was performed. The p19 binds siRNAs with high affinity once the hpRNA is processed by RNase III into short 21–24 nt siRNAs. The siRNA-protein complex was subsequently purified by Ni-NTA affinity chromatography followed by anion-exchange chromatography (Huang et al., 2013).

Encapsulation of dsRNA has been developed as a method to increase yields of dsRNA in microbial host species, and to protect the product from degradation in the application environment. A platform using bacterial minicells for the production and encapsulation of dsRNA has been developed under the name AgriCell technology by AgroSpheres (www.agrospheres.com). This technology involves non-GMO bioparticles produced during the fermentation of bacterial cells which encapsulate the dsRNA, providing protection from environmental degradation including heat, UV, microbes and nucleases and therefore has potential benefits for the endogenous delivery and stable release of the dsRNA. Using this technology dsRNA was manufactured at a yield of 100 mg/L and shown to trigger an RNAi effect in *Botryotinia fuckeliana*, and to inhibit grey mould infection in glasshouse-grown strawberries (Islam et al., 2021).

The potential for the use of virus like particles (VLPs) for dsRNA delivery to insect pests, and the challenges associated with this method, have been reviewed by Kolliopoulou et al. (2017) and VLPs have been successfully used in order to protect against yellow head virus in shrimp (Sinnuengnong et al., 2018). The technology utilises VLPs that co-assemble in the expression species, specifically non-infectious viral envelopes co-expressed in *E. coli* with the dsRNA and protect dsRNA from nucleases and harsh lysis steps, assisting in the isolation, purification and application of crop protecting agents.

#### Inactivation of microorganisms for application in the field

The production of dsRNA in microorganisms offers alternative routes for foliar application of the dsRNA. The dsRNA can either be purified from the host microorganism or delivered *via* direct foliar application of the microorganism containing the dsRNA itself. However, microorganisms transformed with plasmids for production of dsRNA that target insect mRNAs are GM organisms. Therefore prior to application of the microorganism, strategies have been employed to inactivate the microorganisms. For example, *E. coli* cells expressing insecticidal dsRNA and subsequently heat inactivated, resulted in high mortality of beet armyworm through feeding, and resulted in no detectable bacterial colonies when plated on standard LB agar plates (Kim et al., 2015) demonstrating the organism is not able to reproduce in the environment.

Heat-inactivated yeast expressing a dsRNA insecticide have also been demonstrated to be successful in killing larvae of *Anopheles* mosquitoes, as a possible measure against this common disease vector (Mysore et al., 2017). dsRNA produced in *E. coli* which are subsequently heat-inactivated has also been demonstrated to successfully induce RNAi in fish cells (García et al., 2015) and if uptake into live fish could be achieved this technique may have applications outside of insecticides, such as virus control in fish farming.

RNAi has also been induced successfully in *Aedes* mosquitoes fed *E. coli* containing insecticidal dsRNA that were inactivated and lysed by chlorhexidine (Lopez et al., 2019). In addition, Hashiro et al., evaluated the use of alcohols as a sterilising agent for *C. glutamicum* (Hashiro et al., 2019a) and identified this as a preferred method of microbe inactivation, as opposed to heat sterilisation, previously reported (Zhu et al., 2011). Alcohols were shown to permeate cell membranes and inactivate RNases without disruption and degradation of dsRNA. Microbe membranes were noted to still be partially intact, thus protecting active dsRNA from environmental degradation.

#### Bacterium-mediated RNA interference

An alternative approach, also using microorganisms for production of dsRNA is bacterium-mediated RNA interference. In this system live bacteria expressing dsRNA are applied to and colonise an organism to produce and facilitate the uptake of dsRNA resulting in RNAi effects on the targeted mRNA. Using this approach, an appropriate delivery bacterium is selected in conjunction with a plasmid construct to express the dsRNA similarly to approaches outlined previously. The overall aim is to enable the bacterium to replicate *in vivo*, synthesise specific dsRNA molecules that can be absorbed by the insect gut and induce systemic RNAi, either following secretion by the bacteria or after bacterial cell death and lysis *in insecta*.

Although such approaches have been utilised in other systems, their application in crop plant protection is limited. Such approaches have been demonstrated using two different insect species: *Rhodnius prolixus*, a trypanosome-transmitting assassin bug, and *Frankliniella occidentalis*, the western flower thrip, an invasive agricultural pest (Whitten et al., 2016). RNase III-deficient, dsRNA-expressing strains of *R. rhodnii*–a symbiont of these pest species–were created. The production system included an RNase III mutant with a stably integrated dsRNA expression cassette within the *R. rhodnii* chromosome, and dsRNA expressed from plasmids. In both systems the dsRNA expressing *R. rhodnii* were able to produce sustained systemic silencing. In *F. occidentalis*, RNAi reduction of α-tubulin mRNA was achieved and produced significant mortality in both larvae and adults, which was not observed using heat-inactivated bacteria expressing dsRNA (Whitten et al., 2016).

#### Fungal and algal-mediated RNA interference

In addition to using live bacteria for dsRNA production, the use of live insect-parasitising fungi for production and delivery of insecticidal dsRNA is also a possibility. Since 1995, there have been 26 licences approved for the use of genetically modified fungi as crop protection agents in the USA (Hokanson et al., 2014). This generates an appealing avenue for RNA based crop protection strategies as approval remains a significant hurdle to overcome.

*Dialeurodes citri* is a citrus fruit pest which causes soot disease in citrus crops in many locations globally. Currently, only chemical pesticides are used to control *D. citri* (Yu et al., 2019)*. L. attenuatum*, an entomopathogenic fungus which causes decreased population size in *D. citri*, has been explored as a natural alternative to chemical pesticides. However, application of the wild type fungi only results in low mortality rates and therefore, it has not been successfully implemented as a biological agent. Different strains of *L. attenuatum* were developed to produce dsRNA specific to four of the *C. citri* immune responses, under the control of a constitutive promoter PtrpC (Yu et al., 2019). The fungi produced hpRNA which was subsequently processed by the fungal RNAi machinery into siRNAs. Although siRNAs are not usually effective for RNAi in insects (typically dsRNA >80 bp is required for entry *via* the midgut (Huvenne and Smagghe, 2010; Yu et al., 2013)), in this case the delivery method is not through ingestion but *via* a fungal host. RNAi activity was detected by day three of infection with live fungi producing hpRNA, and mortality was slightly increased in two of the targets (Yu et al., 2019).

In a similar study by Hu et al. (Hu and Xia, 2019), virulence of the locust specific fungus *Metarhizium acridum* was increased by the introduction of dsRNA targeting mRNAs specific to the host’s ATPase subunits F_0_-F_1._ Again, the resulting hpRNA was generated under the control of a strong constitutive fungal promoter PmaGDP (Cao et al., 2012; Hu and Xia, 2019). Production of insecticidal dsRNA in live non-parasitic fungi has also been demonstrated, as in the case of production in live yeast (*see* above). Similarly, genetically modified live algae have also been used to deliver dsRNA to mosquito larvae in an aquatic environment (Kumar et al., 2013). In addition, the green microalgae *Chlamydomonas reinhardtii* was engineered to produce dsRNA targeting the lethal shrimp yellow head virus (YHV) and is considered as “Generally Recognized As Safe (GRAS)” by the US Food and Drug Administration. The microalgae does not produce any endotoxins and infectious agents, and therefore is unlikely to result in associated health risk or environmental contamination (Dauvillée et al., 2010).

### Transgenic production of dsRNA *in planta*

A wide range of studies have shown the feasibility of transgenic crops protecting plants against pests and pathogens (Baum et al., 2007; Mao et al., 2011; Pitino et al., 2011; Zha et al., 2011; Zhang et al., 2015; Malik et al., 2016; Yu et al., 2016; Khalid et al., 2017; Li et al., 2017). Transgenic crop protection has also been reviewed in a number of publications (Talakayala et al., 2020; Chung et al., 2021; Kumari et al., 2022). The main advantage associated with *in planta* dsRNA synthesis (endogenous dsRNA production) in comparison to alternative production systems, is the elimination of downstream processing, with no purification, formulation and/or delivery of the dsRNA or material containing the dsRNA required, thus reducing labour for agricultural workers and consequently reducing costs to the consumer (Zotti and Smagghe, 2015). Research has been undertaken examining the feasibility of protecting crops against a variety of organisms, with particular success in protection against plant viruses. Crops which produce their own dsRNA are efficient at “self-protection,” a highly useful technique used for cultivated crops. Some of the previously reported crops which have increased resistance to viruses are tobacco against TMV (Kalantidis et al., 2002), potato against SPV (Sano et al., 1997) and tomato which showed an 82% increase in resistance to four tomato specific viruses with a single transgene (Bucher et al., 2006).

Other research has focused on the protection of crops against insect pests, the earliest of which was undertaken by Mao et al. (Baum et al., 2007), in which F1 corn plants were genetically engineered to express a dsRNA homologue of the *V-ATPase A* mRNA of the Western corn rootworm (WCR). A cassette synthesising a hairpin RNA homologue of the *V-ATPase A* mRNA was produced, under the control of a constitutive promoter CaMV e35S–a design like that in Figure 4C. The cassette was cloned into a vector which aided in chromosomal insertion of the cassette into the host genome (Baum et al., 2007), resulting in dsRNA generation in all cells of the plant. In the study dsRNA was isolated and imaged, showing the product to be intact hpRNA and Dicer processed siRNAs (21–24 nt in length). As previously mentioned, siRNA is not active for RNAi when ingested by insect pests, and dsRNA of lengths <60 bp have limited uptake (Bolognesi et al., 2012). However, as some hpRNA was available, despite being processed by the host’s RNAi machinery, uptake was achieved by the target pest and RNAi activity was observed, with the insects demonstrating signs of impaired growth and development. Other studies also achieved similar results but none could produce full plant protection or 100% insect mortality (Baum et al., 2007; Mao et al., 2011; Pitino et al., 2011; Zha et al., 2011).

Recently, a number of laboratories have shown that producing dsRNA in a plant’s chloroplast, rather than in its cellular cytoplasm, results in improved delivery of dsRNA (Jin et al., 2015; Zhang et al., 2015; Bally et al., 2016). Plastids are plant organelles formally believed to have been freely living prokaryotic cyanobacteria, and therefore lack RNAi machinery. It was hypothesised that dsRNA may therefore accumulate in plastids, for example in transplastomic potato plants with dsRNA targeting the Colorado potato beetle (CPB) (He et al., 2020). Three forms of dsRNA were produced in tobacco plastids to assess yield and homogeneity: linear dsRNA, hairpin RNA, and a linear RNA with hairpin protective ends. Constructs for the linear and hairpin forms of the dsRNA were identical to those previously described (*see* Figures 4B, C respectively) except for the substitution of promoter type. The novel hairpin protected dsRNA (hppRNA) was synthesised in a similar manner to linear dsRNA, apart from a small alteration by way of insertion of small self-complimentary inverted repeats at either end of the targeting region. These are predicted to self fold into small hairpins which protect linear dsRNA from RNases.

Accumulation of dsRNA was achieved in all three cases, with linear dsRNA having the highest yields. Zhang et al., subsequently produced transplastomic potato crops that showed dsRNA accumulation levels in leaves of 0.4% of the total cellular RNA (Zhang et al., 2015). This level of expression was enough to provide full plant protection from the CPB larvae when targeting *β-actin* (an essential cytoskeleton protein) causing 100% mortality after 5 days of insect feeding. These studies also highlight the importance of producing large amounts of long dsRNAs to achieve efficient protection.

The development of GM crops is a long and costly process, and engineered plants may become redundant in a short period of time (Khajuria et al., 2018) with the ever growing issue of pest resistance. Additionally, expression of dsRNA in transgenic plants is less flexible and adaptable than other RNA-based control methods, and hampered by political issues surrounding GM crops (Borel, 2017). There is strong resistance to the introduction of GM crops from public groups and the media, in spite of the ever-growing support from scientific research (Zhang et al., 2015). Legislation also holds back the development of transgenic crops, particularly in Europe where growth of genetically modified crops is restricted (Papademetriou, 2015). A further limitation of genetically engineered crops is well described by the control of the Canadian pine beetle, which threatens the pine forests of Canada. The beetles’ main food source is well-established trees, and thus genetically engineering plants would not prove an effective biocontrol measure (Keeling et al., 2013).

2017 was a landmark year for dsRNA crop protection as the first commercially available GMO crop was approved by the FDA with Monsanto and Dow’s SMARTSTAX PRO maize (Head et al., 2017). The crop produces an active dsRNA targeting the western corn rootworm mRNA of *Snf7.* Furthermore, studies were also approved to investigate quality enhancement of apple and potato crops which express dsRNA for the regulation of endogenous proteins (Waltz, 2015; Baranski et al., 2019).

## Discussion

The development of dsRNA based biocontrols for effective pest management strategies, requires the production of large quantities of dsRNA combined with suitable delivery methods to ensure RNAi is effectively triggered in the target pest. For large field-scale management of crop pests and pathogens, large quantities of dsRNA would be required (>1000 kg of dsRNA). Previous estimations have suggested up to 10 g of dsRNA per hectare (Zotti et al., 2018), although this amount may vary depending on the target’s sensitivity to trigger RNAi *via* oral uptake of dsRNA and its capacity for systemic RNAi. Such large scale manufacturing at appropriate cost is challenging and will be difficult to achieve using *in vitro* dsRNA transcription systems, which exhibit a minimum cost of $100 per g of dsRNA (Zotti et al., 2018). However recent advances in the manufacturing of mRNA vaccines have demonstrated the scalability of IVT for the large-scale manufacturing of RNA using such approaches (Sahin et al., 2020). Large scale microbial fermentation methods offer the potential for more economical large scale biomanufacturing of dsRNA and several industrial companies are currently developing low-cost, large-scale manufacturing platforms for the production of dsRNA typically <$1/g (Zotti et al., 2018).

*In vitro* transcription is a rapid, versatile method capable of producing high purity dsRNA which is particularly useful for research focussing on the development of new dsRNA based biocontrols and laboratory scale research studies. Large-scale IVT is limited but commercially available and capable of generating Gram to kilogram quantities of 100–800 bp dsRNA (Cagliari et al., 2018). Large scale IVT typically uses convergent T7 DdRp promoters flanking the target sequence. However, limited yields and lengthy production times for industrial scale production potentially limit its agricultural implementation. Cell-free systems offer an alternative scalable production method with the advantages of low cost and high yield, although currently this requires specialist commercial production. Microbial systems offer an alternative approach for production of dsRNA which is versatile and scalable. Moreover *E. coli* is currently widely used in high cell density fermentation processes for industrial recombinant protein production and is also able to achieve high dsRNA yields. In addition, alternative new microbial hosts are emerging as alternative production systems which will provide new opportunities and strategies for the *in vivo* production of exogenous dsRNA based biocontrols economically at scale, for example *C. glutamicum* which recently achieved yields of over 1 g/L dsRNA (Hashiro et al., 2021).

Bacterially mediated RNAi could provide an alternative approach for the production and delivery of dsRNA, enabling a semi-permanent RNAi silencing effect to be induced by exogenous application. Use of a plant symbiote microorganism offers additional benefits compared to topical application of dsRNA, as the symbiote can colonise the host plant, synthesising protective dsRNA *in situ*. However, such approaches are limited by the availability of an appropriate delivery bacterium for which suitable dsRNA expression cassettes and vectors have been developed. It is also presently unclear how bacteria might transmit silencing RNA to the plant. Furthermore, such approaches will require the release of GM organisms into the environment and raise potential issues of public acceptance.

Transgenic organisms for the production of endogenous dsRNA possess a number of advantages over non-transformative methods as well as a few drawbacks. Fungal production is in its infancy when compared to other methods but with further optimisation–for example, the knockout of RNAi machinery within the fungi–there is potential that transgenic fungi may play a part in the future of bio-pesticides. Additionally, the approval of a number of licences for the use of transgenic fungi by regulatory bodies shows promise for further approval for crop protection. *In planta* production, again shows promise for crop protection with a number for studies highlighting its key benefits. Expression in transgenic plants works for commercial crops such as corn. However, such approaches are expensive, time consuming, less flexible and hampered by issues surrounding GM crops.

Beyond the challenges associated with the production method, there are also challenges surrounding the delivery of dsRNA biocontrols and the variability of RNAi efficacy between different target species. Different insect orders and species show variation in RNAi efficacy, with orders such as Coleoptera and Hemipterea generally showing good RNAi efficacy. In contrast, many Lepidoptera demonstrate poor RNAi efficacy. This variation is due to factors such as insect nuclease potency and upregulation (Guan et al., 2018), physiological pH prone to causing RNA hydrolysis (Shukla et al., 2016), and differences in dsRNA uptake and subsequent intracellular transport (Shukla et al., 2016). As these factors can often be overcome by varying the dsRNA delivery method, and certain delivery methods are also more or less amenable to particular production methods, both production and delivery will have to be dovetailed in order to achieve efficient dsRNA biocontrols against certain species of pest insect.

Whilst research in production of dsRNA biocontrols has resulted in many publications few products have been commercialised. The plant-incorporated protectant product MON-87411 is a genetically engineered maize that among other transgenes produces a dsRNA matching the sequence of part of the *snf7* essential gene from Western corn rootworm (*Diabrotica virgifera virgifera*). MON-87411 has been registered for cultivation in the US and elsewhere since 2015 (Firko, 2015). The grain product of this crop has been registered for food and feed uses within the European Union (Naegeli et al., 2019). To date, no other dsRNA-based GM crop has been commercialized.

No spray applied RNA-based biocontrols have been commercialized. However, Greenlight Biosciences indicates that it plans to register a treatment for Colorado potato beetle during 2022 with first sales planned in the US during 2023. One RNA-based biocontrol has been given initial registration to protect honeybees from Israeli acute paralysis virus (IAPV). The registration holder, Beeologics was acquired by Monsanto, subsequently acquired by Bayer who sold the technology to GreenLight. Research data suggested that this technology was effective in the controlling IAPV in commercial beehives (Hunter et al., 2010).

## Conclusion

In conclusion, dsRNA based biocontrols have the potential to provide a species-selective and sustainable insect management strategy that overcomes many current issues associated with chemical pesticides. However, research on the large-scale manufacturing and mass delivery of dsRNA insecticides is in its infancy, and all potential methods present challenges. Different methods may also be more suited to particular target insects or application environments, and have additional challenges related to product characterisation, quantification, quality control and regulatory approval that need to be addressed. Furthermore, there is unlikely to be a universal method that is effective for control of all conceivable target species, and production and delivery methods will have to be tailored accordingly. However, a number of methods presented here demonstrate potential for large scale commercial production of dsRNA based biocontrols as sustainable alternatives to chemical pesticides.
